# DCE-MRI assessment of the effect of Epstein-Barr virus-encoded latent membrane protein-1 targeted DNAzyme on tumor vasculature in patients with nasopharyngeal carcinomas

**DOI:** 10.1186/1471-2407-14-835

**Published:** 2014-11-18

**Authors:** Wei-Hua Liao, Li-Fang Yang, Xiao-Yu Liu, Gao-Feng Zhou, Wu-Zhong Jiang, Bob-Lei Hou, Lun-Quan Sun, Ya Cao, Xiao-Yi Wang

**Affiliations:** Department of Radiology, Xiangya Hospital, Central South University, Changsha, Hunan China; Cancer Research Institute, Key Laboratory of Chinese Ministry of Education, Central South University, Changsha, Hunan China; Center for Molecular Imaging, Central South University, Changsha, Hunan China; MRI Division, Ganzhou People’s Hospital, Ganzhou, Jiangxi 341000 China; Department of Oncology, Xiangya Hospital, Central South University, Changsha, Hunan China; Center of Advanced Imaging, Department of Radiology, West Virginia University, Morgantown, WV 26506 USA; Center for Molecular Medicine, Central South University, Changsha, Hunan China

**Keywords:** Nasopharyngeal neoplasm, Epstein-Barr virus encoded latent membrane protein-1, Magnetic resonance imaging, Dynamic contrast-enhanced magnetic resonance imaging, Molecular therapy, DNAzyme

## Abstract

**Background:**

EBV-encoded latent membrane protein 1 (EBV-LMP1) is an important oncogenic protein for nasopharyngeal carcinoma (NPC) and has been shown to engage a plethora of signaling pathways. Correspondingly, an LMP1-targeted DNAzyme was found to inhibit the growth of NPC cells both *in vivo* and *in vitro* by suppressing cell proliferation and inducing apoptosis. However, it remains unknown whether an LMP1-targeted DNAzyme would affect the vasculature of NPC. Dynamic contrast-enhanced magnetic resonance imaging (DCE-MRI) has been applied in the clinical trials of anti-angiogenic drugs for more than ten years, and *K*^trans^ has been recommended as a primary endpoint. Therefore, the objective of the current study was to use DCE-MRI to longitudinally study the effect of an EBV-LMP1-targeted DNAzyme on the vasculature of patients with NPC.

**Methods:**

Twenty-four patients were randomly divided into two groups: a combined treatment group (radiotherapy + LMP1-targeted DNAzyme) and a radiotherapy alone group (radiotherapy + normal saline). DCE-MRI scans were conducted 1 ~ 2 days before radiotherapy (Pre-RT), during radiotherapy (RT 50 Gy), upon completion of radiotherapy (RT 70 Gy), and three months after radiotherapy (3 months post-RT). Parameters of vascular permeability and intra- and extravascular volumes were subsequently obtained (e.g., *K*^trans^, *k*_ep_, *v*_e_) using nordicICE software.

**Results:**

Both *K*^trans^ and *k*_ep_ values for NPC tumor tissues decreased for both groups after treatment. Moreover, a statistically significant difference in *K*^trans^ values at the pre-therapy and post-therapy timepoints emerged earlier for the combined treatment group (RT 50 Gy, *P* =0.045) compared to the radiotherapy alone group (3 months post-RT, *P* = 0.032). For the *k*_ep_ values, the downward trend observed for both the combined treatment group and the radiotherapy alone group were similar. In contrast, *v*_e_ values for all of the tumor tissues increased following therapy.

**Conclusions:**

The EBV-LMP1-targeted DNAzyme that was tested was found to accelerate the decline of K^trans^ values for patients with NPC. Correspondingly, the LMP1-targeted DNAzyme treatments were found to affect the angiogenesis and microvascular permeability of NPC.

**Trial registration:**

ClinicalTrials.gov: NCT01449942. Registered 6 October 2011.

## Background

Nasopharyngeal carcinoma (NPC) is a common malignancy in southern China. Currently, radiation therapy is the preferred treatment for this disease. With the emergence of intensity-modulated radiation therapy (IMRT), local control rates for tumors have significantly increased [1]. However, increased radiosensitivity, reduced radiation doses, and improved quality of life for NPC patients remain active areas of research. In particular, receptor-targeted molecular therapy, as well as the identification of key genes, regulatory elements, and kinases in NPC cells, are being studied.

Epstein-Barr virus (EBV) has been implicated in the pathogenesis of NPC, and EBV-encoded latent membrane protein 1 (EBV-LMP1)) has been detected in 90% of NPC clinical samples [2]. EBV-LMP1 is an important oncogenic protein that has been shown to engage a plethora of signaling pathways that involve nuclear factor kappa B (NFκB), c-Jun N-terminal kinase (JNK), c-Jun activator protein 1 (AP1), mitogen A activated protein kinase (p38a MAPK), activating transcriptional factor (ATF), Janus kinase (JAK) signal transducers, and activators of transcription protein (STAT). Moreover, constitutive activation of these pathways appears to be central to the ability of LMP1 to induce multiple morphological and phenotypic alterations [3]. For example, EBV-LMP1 is involved in the transformation, proliferation, apoptosis, and differentiation of NPC cells, and has also exhibited a positive effect on tumor angiogenesis by directly up-regulating expression of vascular endothelial growth factor (VEGF) via the Stat 3 transcription factor [4]. Therefore, it has been hypothesized that blocking of EBV-LMP1 represents a novel targeted molecular therapy for NPC [5].

Deoxyribozymes (DNAzymes) are single-stranded DNA fragments that have been selected to have catalytic functions and ribozyme activity using *in vitro* molecular evolution techniques. DNAzymes have the ability to cleave RNA with high efficiency [6], and therefore, are able to inhibit gene expression at the mRNA level and regulate expression of target proteins [7, 8]. Correspondingly, DNAzymes are potentially applicable to gene inactivation strategies [9, 10]. Previously, a 33-mer oligonucleotide LMP1-targeted DNAzyme containing three phosphorothioate linkages at its 5’ and 3’ ends was developed to specifically target *LMP1* mRNA [11]. Down-regulation of LMP1 expression using this LMP1-targeted DNAzyme was found to inhibit the growth of NPC cells both *in vivo* and *in vitro* by suppressing cell proliferation and inducing apoptosis [2, 12]. Moreover, in a recent study, LMP1-targeted DNAzyme was found to enhance the radiosensitivity of LMP1-positive NPC cells by inhibiting telomerase activity [13].

Conventionally, prospective, random, and well-controlled double-blind trials have been the “gold standard” for evaluating drug safety and effectiveness. Unfortunately, however, this method has also resulted in the soaring costs associated with medical innovations. Consequently, it has been proposed that biomarkers could be used to provide initial evidence regarding drug efficacy and safety. In recent years, with advances in imaging technology, imaging biomarkers have been increasingly utilized to assess drug efficacy. Furthermore, newer imaging technologies have been able to provide functional information in addition to structural information for several diseases [14].

Dynamic contrast-enhanced magnetic resonance imaging (DCE-MRI) uses fast T1-weighted sequences to assess changes in signal intensity before, during, and after the intravenous administration of contrast agent (CA). The dynamic contrast images obtained are then used to quantitate parameters which characterize tumor microcirculation. For example, the volume constant for the transfer of CA from the plasma to the extravascular extracellular space (e.g., *K*^trans^ minute^-1^) represents an important parameter, and is primarily related to blood perfusion and microvessel permeability [15]. Over the past 10 years, DCE-MRI and extracted kinetic parameters have been applied to phase I and phase II clinical trials of anti-angiogenic drugs and vascular disrupting agents, with *K*^trans^ generally recognized as a marker of tumor blood flow and permeability [15–17]. In addition, *K*^trans^ has been recommended as a primary endpoint for anti-cancer treatment trials by the U.S. National Cancer Institute [18].

Although EBV LMP1 has been shown to promote tumor angiogenesis [4], it remains unknown whether an LMP1-targeted DNAzyme affects NPC vasculature. In the present study, an LMP1-targeted DNAzyme was injected locally into NPC tissues in conjunction with radical radiation therapy and DCE-MRI was used to evaluate the efficacy of this treatment for NPC patients. To the best of our knowledge, this study is the first to examine the effects of an LMP1-targeted DNAzyme using DCE-MRI *in vivo*.

## Methods

### Preparation of EBV LMP1-targeted DNAzyme

The EBV LMP1-targeted DNAzyme tested was produced by Oligos Etc, Inc. (Wilsonville, OR, USA) under Good Manufacturing Practice conditions. The efficacy, toxicity, and plasma pharmacokinetics of this LMP1-targeted DNAzyme was previously evaluated in mice prior to clinical study [2]. Based on the results of these studies, this LMP1-targeted DNAzyme was found to be safe and well-tolerated.

### Patients and grouping

A total of 24 patients with histologically confirmed NPC were enrolled in this study and received treatment at the Xiangya Hospital. This cohort included 18 males and 6 females who ranged in age from 31–64 years (Table 1). Poorly differentiated squamous cell carcinoma was confirmed by biopsy in all 24 cases, while EBV-LMP1 positive expression was confirmed with immunohistochemical techniques. In addition, 17 cases involved cervical lymph node metastasis, while distant metastases were not detected in any of the cases.

**Table 1:** **Patient characteristics**

Patient no.	Gender	Age (y)	Tumor stage	Treatment group
1	F	36	II	Combined treatment
2	M	44	II	Radiotherapy alone
3	M	63	II	Combined treatment
4	F	32	III	Combined treatment
5	M	56	III	Combined treatment
6	F	45	IVa	Radiotherapy alone
7	M	40	III	Radiotherapy alone
8	F	44	IVa	Combined treatment
9	M	48	II	Combined treatment
10	M	60	III	Combined treatment
11	M	64	III	Radiotherapy alone
12	M	55	III	Combined treatment
13	M	43	IVa	Radiotherapy alone
14	M	59	II	Combined treatment
15	F	56	II	Radiotherapy alone
16	M	37	II	Combined treatment
17	F	37	II	Combined treatment
18	M	41	II	Radiotherapy alone
19	M	52	II	Combined treatment
20	M	41	II	Radiotherapy alone
21	M	31	II	Combined treatment
22	M	51	II	Combined treatment
23	M	52	II	Radiotherapy alone
24	M	62	II	Radiotherapy alone

### Protocol design

Twenty-four patients were randomly and double-blindly divided into two groups: a combined treatment group (n = 14) or a radiotherapy alone group (n =10) (Table 1). The randomization of patients was not ideal since six patients (1 in the combined treatment group and 5 in the radiotherapy alone group) rejected DCE-MRI. For both treatment groups, a Varian 2100C/D linear accelerator was used for radiotherapy.

The combined treatment group received radiotherapy plus an injection of the LMP1-targeted DNAzyme in saline (6 mg/0.1 ml) into each tumor. Regarding the latter, patients received local anesthesia and an epical endoscope was used to guide the injections. The injections were performed twice a week for five weeks, resulting in a total volume of 1 ml LMP1-targeted DNAzyme administered to each tumor. For radiotherapy, a 2 Gy dose was administered five times a week for seven weeks, resulting in a total dose of 70 Gy.

For the radiotherapy alone group, the program and dose of radiotherapy were the same as that described for the combined treatment group. In addition, the patients received local anesthesia for injections of saline (0.1 ml each) that were administered using an epical endoscope. Injections were performed twice a week for five weeks, resulting in a total volume of 1 ml saline administered to each tumor.

This study was conducted in accordance with the International Conference on Harmonization-Good Clinical Practice. Approval for this study was obtained from the Human Ethics Committee of the Xiangya Hospital, Central South University, and written consent was obtained from each participant. Approval was also obtained from the Ministry of Science and Technology.

### MRI protocol

All patients underwent conventional MRI and DCE-MRI scans 1 ~ 2 days prior to the start of radiotherapy (Pre-RT). Subsequent scans were performed during the radiotherapy treatment period after a radiation dose of 50 Gy had been achieved (RT 50 Gy), at the end of radiotherapy when a total radiation dose of 70 Gy had been applied (RT 70 Gy), and three months after radiotherapy (3 months post-RT). MRI was performed using a 1.5 T system (MAGNETOM Sonata, Siemens, Erlangen, Germany).

#### Conventional MRI

Unenhanced axial T1-weighted (repetition time/echo time [TR/TE] 450/10 ms, field of view [FOV] 230 × 230 mm, section thickness 5 mm, intersection gap 1.5 mm, 19 sections) and axial T2-weighted (TR/TE 4200/98 ms) images were obtained. Contrast-enhanced and fat-suppression T1-weighted images (TR/TE 450/10) were obtained following the DCE-MRI scans.

#### DCE-MRI

DCE-MRI was acquired using a TurboFLASH sequence before, during, and after an intravenous injection of CA was made. Ten axial sections were selected from throughout the tumor on the basis of T2-weighted imaging. Parameters were as follows: TR/TE 199 ms/1.05 ms, inverse time 100 ms, FOV 260 mm × 210 mm, matrix 128 × 96, flip angle 20°, section thickness 6 mm, intersection gap 1.2 mm, time resolution 4 s. A series of 90 multisection sets were acquired in 360 s. At the end of the sixth acquisition, a standard dose (0.2 mmol/kg body weight) bolus of CA (Magnevist, Bayer Schering Pharma AG, Berlin, Germany) was injected via the antecubital vein at a rate of 4 ml/s using a CA power injector (OptiStarTM LE, Mallinckrodt), followed by a 20 ml bolus of saline at a rate of 4 ml/s.

### Image postprocessing

The original DCE-MRI data were transferred to an independent workstation and processed using a nordicICE software package (Nordic Image Control and Evaluation Version 2.3.6; Nordic Imaging Lab, Bergen, Norway). First, the arterial input function (AIF) was automatically obtained from a region of interest (ROI) drawn on the internal carotid artery located in close proximity to the tumor. Secondly, based on Tofts-kermode two compartment kinetic modeling theory [19], deconvolution of the tissue response curves (dynamic curves for all pixels) was performed using the AIF according to Keunen [20]. Qualitative, as well as quantitative, maps of several parameters (including *K*^trans^, *k*_ep_, *v*_e_) related to vascular permeability and intra- and extravascular volumes were subsequently obtained. To calculate the *K*^trans^ for each tumor, a ROI (with a diameter of 9-10 pixels, or 60-70 mm^2^) was placed on the tumor parenchyma where the *K*^trans^ value was the highest. For the same ROI, the other parameters were calculated. *K*^trans^ represents the rate constant for the transfer of CA from plasma to the extravascular extracellular space (EES). In addition, *k*_ep_ is the rate constant for the transfer of CA from EES to plasma, and *v*_e_ is the CA distribution volume. These parameters are related as follows: *v*_e_ = (*K*^trans^/*k*_ep_).

### Safety evaluation

Adverse events were reviewed at each patient visit. Blood samples were also collected from patients prior to radiotherapy, during radiotherapy (after 50 Gy), and three months after radiotherapy. Analyses of blood chemistry, as well as liver and renal function, were evaluated. Impairment of the skin, mucous membranes, and salivary gland are common characteristics of radiation therapy, and their incidence was analyzed using the Wilcoxon rank sum test.

### Statistical analysis

All data are expressed as the mean ± standard deviation (SD), and confidence intervals are included in the figures. Using Student’s *t*-test, values between the different groups were compared. In addition, the values at various timepoints were compared for the same group using the Student-Newman-Keuls (SNK)-q test. A *P*-value less than 0.05 was considered statistically significant. Statistical analyses were performed using SPSS (version 17.0; SPSS Inc., Chicago, IL, USA).

## Results

**Figure 1:**
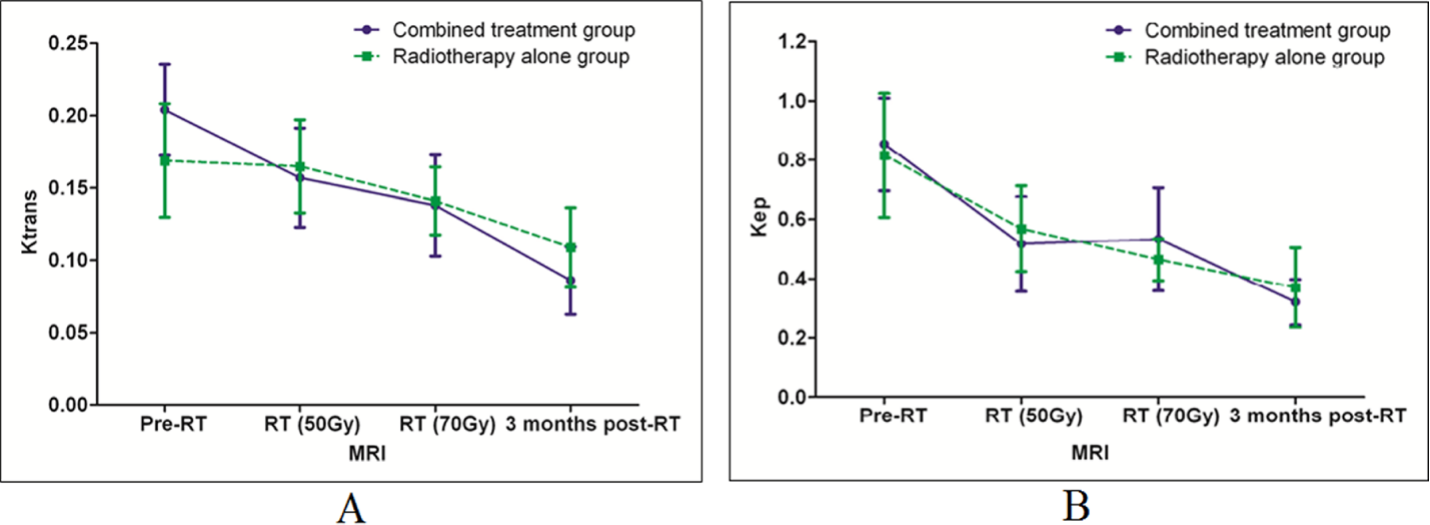
***K*** ^**trans**^ **values (A) and** ***k***_**ep**_ **values (B) their associated 95% CIs are shown for the Pre-RT, RT (50 Gy), RT (70 Gy), and 3 months post-RT timepoints for the combined treatment group and the radiotherapy alone group.** The *K* ^trans^ values for the NPC tumor tissues decreased in both groups. Furthermore, statistically significant differences in the pre-therapy and post-therapy *K* ^trans^ values emerged earlier in the combined treatment group (RT 50 Gy) compared to the radiotherapy alone group (3 months post-RT) **(A)**. In both groups, the *k*_ep_ values declined gradually following therapy **(B)**.

**Figure 2:**
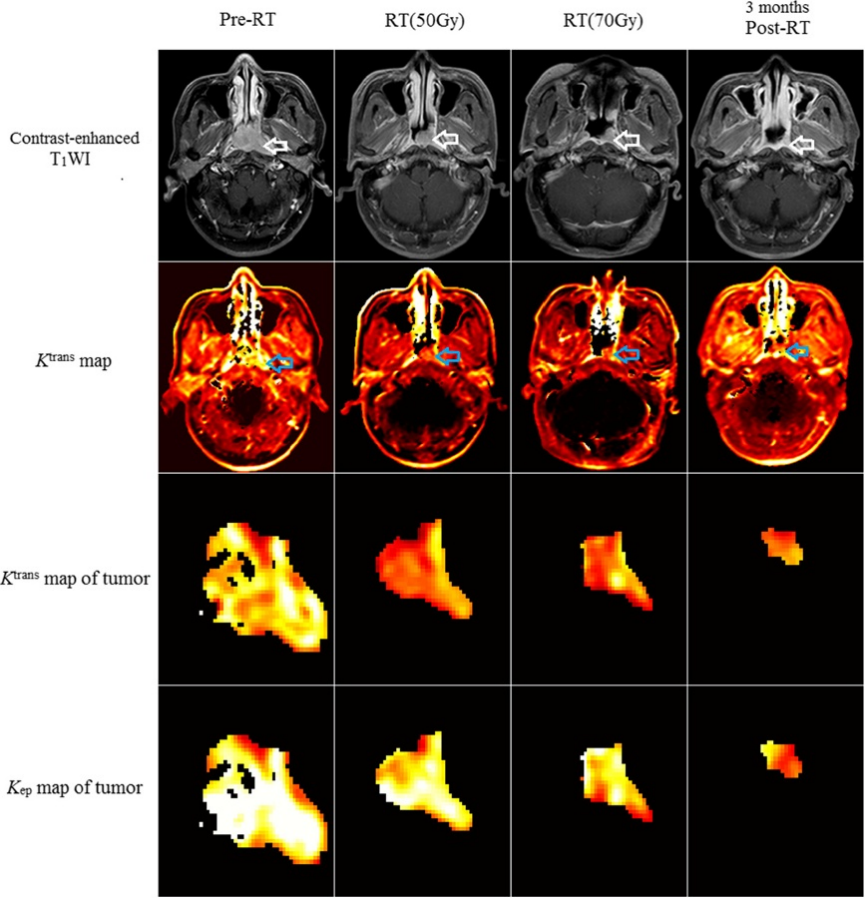
**The change of** ***K*** ^**trans**^ **values (A) and** ***k***_**ep**_ **values (B) and their associated 95% CIs for different timepoints for the combined treatment group and the radiotherapy alone group.** The *K* ^trans^ value of the tumor for pre-RT, RT 50 Gy, RT 70 Gy and 3 months post-RT timepoint is 0.2391, 0.1684, 0.1623, 0.1012 (minute^-1^) respectively, which demonstrates a significant decrease in the *K* ^trans^ at the RT 50 Gy timepoint, and the *k*_ep_ value is 1.2913, 0.7172, 1.0940, 0.4545 (minute^-1^) respectively.

**Figure 3:**
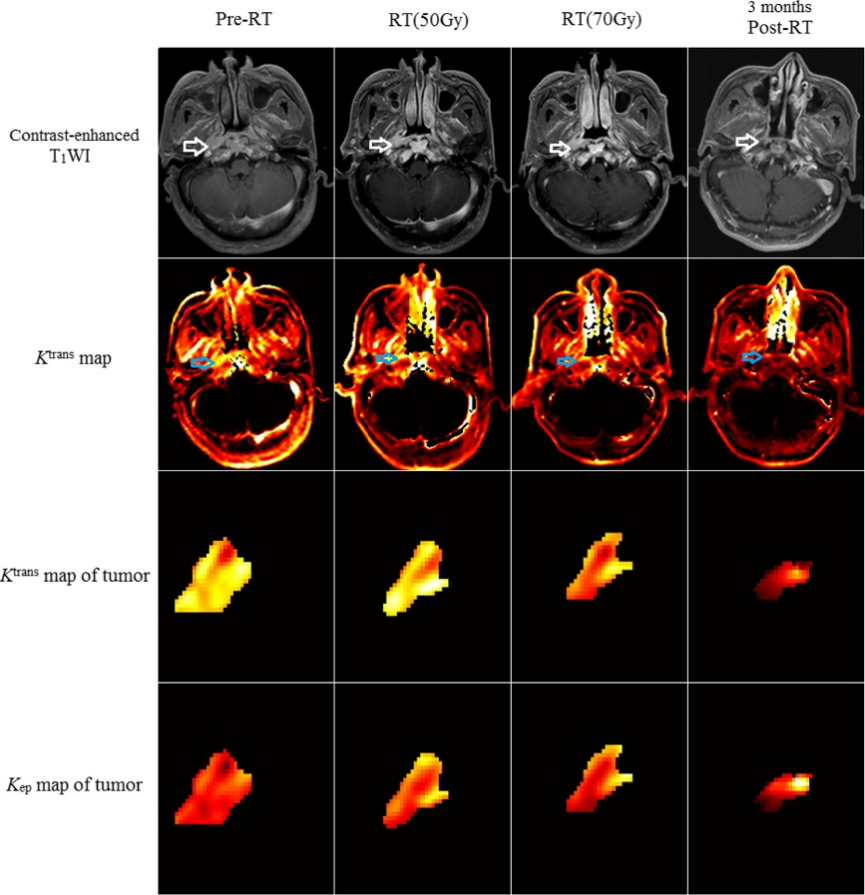
**Patient 20 of the radiotherapy alone group (radiotherapy + normal saline).** The *K* ^trans^ value of the tumor for pre-RT, RT 50 Gy, RT 70 Gy and 3 months post-RT timepoint is 0.1891, 0.1622, 0.1429, 0.0633 (minute^-1^) respectively, which shows a significant decrease in the *K* ^trans^ at 3 months post-RT, and the *k*_ep_ value is 0.5338, 0.3982, 0.3570, 0.0900 (minute^-1^) respectively.

To validate whether DCE-MRI can monitor the therapeutic efficacy of this treatment approach for NPC patients, parameters of vascular permeability and intra- and extravascular volumes were subsequently obtained (including *K*^trans^, *k*_ep_, *v*_e_). Prior to radiotherapy, there were no statistically significant differences in the *K*^trans^ for the tumor tissues of the combined treatment group versus the tumor tissues of the radiotherapy alone group (*t*-test, *P* =0.175). However, for both groups, the 95% confidence interval (CI) of the *K*^trans^ values declined gradually from the Pre-RT timepoint to the 3 months post-RT timepoint (Figure 1A). Moreover, for the combined treatment group, statistically significant differences in the *K*^trans^ values were observed between the Pre-RT timepoint and the RT (50 Gy), RT (70 Gy), and 3 months post-RT timepoints. In particular, the latter values were lower than the *K*^trans^ value for the Pre-RT timepoint. In contrast, the radiotherapy alone group exhibited no significant differences in the *K*^trans^ values between the Pre-RT and the RT (50 Gy) and RT (70 Gy) timepoints. However, there was a statistically significant difference between the *K*^trans^ values for the Pre-RT and 3 months post-RT timepoints for the radiotherapy alone group (Table 2). Representative *K*^trans^ maps are shown in Figures 2 and 3.

**Table 2:** **Comparison of** *K* ^trans^ **values for different timepoints for the two groups**

Timepoints	Combined treatment group			Radiotherapy alone group		
	n	Mean ± SD	***P***-value	n	Mean ± SD	***P***-value
Pre-RT	14	0.2048 ± 0.0606		10	0.1693 ± 0.0619	
RT (50 Gy)	12	0.1573 ± 0.0605	0.045^*^	10	0.1652 ± 0.0518	0.8231^*^
RT (70 Gy)	14	0.1382 ± 0.0671	0.004^**^	9	0.1414 ± 0.0363	0.4262^**^
3 months post-RT	12	0.0862 ± 0.0413	0.000^***^	9	0.1096 ± 0.0424	0.0323^***^

Comparison of *K* ^trans^ values for the Pre-RT, RT (50 Gy), RT (70 Gy), and three months post-RT timepoints for the combined treatment group and the radiotherapy alone group.

^*^Comparison between Pre-RT and RT (50 Gy); ^**^Comparison between Pre-RT and RT (70 Gy); ^***^Comparison between Pre-RT and 3 months post-RT.

There were no statistically significant differences in the *k*_ep_ values for tumor tissues between the combined treatment group and the radiotherapy alone group before radiotherapy (*t*-test, *P* =0.770). However, for both groups, the *k*_ep_ values gradually declined following therapy. The 95% CI for the k_ep_ values are shown in Figure 1B. When *k*_ep_ values were compared for the four timepoints, statistically significant differences were observed between the Pre-RT timepoint and the RT (50 Gy), RT (70 Gy), and 3 months post-RT timepoints for the combined treatment group, and the radiotherapy alone group. In particular, the latter values were lower than the *k*_ep_ value for the Pre-RT timepoint (Table 3). Representative *k*_ep_ maps are shown in Figures 2 and 3.

**Table 3:** **Comparison of** ***k***_**ep**_ **values for different timepoints for the two groups**

Timepoints	Combined treatment group			Radiotherapy alone group		
	n	Mean ± SD	***P***-value	n	Mean ± SD	***P***-value
Pre-RT	14	0.8550 ± 0.2978		10	0.8163 ± 0.3398	
RT (50 Gy)	12	0.51672 ± 0.2845	0.004^*^	10	0.5688 ± 0.2337	0.027^*^
RT (70 Gy)	14	0.5332 ± 0.3347	0.003^**^	9	0.4644 ± 0.1087	0.003^**^
3 months post-RT	12	0.3216 ± 0.1339	0.000^***^	9	0.3703 ± 0.2051	0.000^***^

Comparison of *k*_ep_ values for the Pre-RT, RT (50 Gy), RT (70 Gy), and three months post-RT timepoints for the combined treatment group and the radiotherapy alone group. ^*^Comparison between Pre-RT and RT (50 Gy); ^**^Comparison between Pre-RT and RT (70 Gy); ^***^Comparison between Pre-RT and 3 months post-RT.

Statistically significant differences were observed between the *v*_e_ values for the Pre-RT timepoint and the RT (50 Gy), RT (70 Gy), and 3 months post-RT timepoints for all tumor tissues. Furthermore, the latter values were higher than the *v*_e_ value for the Pre-RT timepoint (Table 4).

**Table 4:** **Comparison of the** *v*_e_ **values for the Pre-RT, RT (50 Gy), RT (70 Gy), and 3 months post-RT timepoints for all of the NPC tissues examined**

Timepoints	n	***v***_e_	
		Mean ± SD	***P***-value
Pre-RT	24	0.2359 ± 0.0754	
RT (50 Gy)	22	0.3316 ± 0.1360	0.009^*^
RT (70 Gy)	23	0.3195 ± 0.1327	0.020^**^
3 months post-RT	21	0.3138 ± 0.1316	0.033^***^

^*^Comparison between Pre-RT and RT (50 Gy); ^**^Comparison between Pre-RT and RT (70 Gy); ^***^Comparison between Pre-RT and 3 months post-RT.

Conventional MRI was performed for all of the participants and was accompanied by DCE-MRI. Enhancement of the tumor parenchyma was observed in both contrast-enhanced and fat-suppression T1-weighted images. The majority of the tumor lesions exhibited a reduction in tumor volume rapidly after treatment. Moreover, two lesions in the combined treatment group and one lesion in the radiotherapy alone group disappeared completely three months after the patients received radiotherapy. Tumor volumes were measured from contrast-enhanced and fat-suppression T1-weighted images by two specialists (an oncologist and a radiologist) using the Varian Eclipse program (Soma Vision). For the combined treatment group, the mean tumor shrink rate 3 months post-RT was significantly higher than that for the radiotherapy alone group (97.78 ± 5.81% and 87.78 ± 15.20%, respectively; *P* = 0.038).

There were no adverse events that could be attributed to LMP1-targeted DNAzyme injections. Furthermore, analyses of white blood cell number, hemoglobin concentration, platelet number, and lymphocyte cell number showed no significant differences between the combined treatment group and the radiotherapy alone group. There were also no significant differences in liver or renal function, or in impairment of skin, mucous membranes, or salivary gland, between the two treatment groups.

## Discussion

Studies have shown that DCE-MRI can be used to monitor the efficacy of various treatments and to predict response to treatment. In particular, this has been demonstrated for neoadjuvant chemotherapy and radiation therapy for bladder cancer, breast cancer, and osteosarcomas [21, 22]. Over the past 10 years, studies have also shown that DCE-MRI and extracted kinetic parameters can be used as an *in vivo* cancer imaging tool for the diagnosis, monitoring of treatment effect, and evaluation of anti-cancer drugs. Correspondingly, this method has been applied to phase I and phase II clinical trials of anti-angiogenic drugs and vascular disrupting agents [23, 24]. In particular, *K*^trans^ is currently recognized as a general marker of tumor blood flow [15–17], and has been recommended as a primary endpoint for an anti-cancer treatment trial conducted by the U.S. National Cancer Institute [18]. DCE-MRI has been widely used in the development of anti-angiogenic drugs, and has detected the efficacy of drugs earlier than conventionally observed changes in tumor volume. This is particularly beneficial for the selection of individualized patient treatment plans for patients that are diagnosed in the early stages of disease [25]. In a summary by O'Connor et al. describing their experience with DCE-MRI for the early clinical development of vascular-directed anticancer therapies over the past decade, they demonstrated that perfusion imaging provides unique information regarding the vascular properties of tumors, and for tumor responses to antiangiogenic agents and VDAs in pre-clinical and early clinical studies [15]. DCE-MRI has also been used as a biomarker for certain chemotherapy drugs in order to separate the biological effects of chemotherapy from those related to angiogenesis inhibitors [26].

To investigate the effects of an EBV LMP1-targeted DNAzyme on NPC vasculature in the present study, two methods were compared (e.g., radiation therapy in combination with a LMP1-targeted DNAzyme and radiation therapy alone) using DCE-MRI. It was observed that the *K*^trans^ values for NPC tumor tissues decreased for both treatment groups over the course of treatment. *K*^trans^ is a factor that accounts for the complex functions of blood flow, endothelial surface area, and endothelial permeability. However, it can also have multiple physiologic interpretations depending on the balance between capillary permeability and blood flow in a tissue. In high-permeability situations (where flux across the endothelium is flow limited), *K*^trans^ is equal to the blood plasma flow per unit volume of tissue. Conversely, for low permeability conditions where tracer flux is permeability limited, the *K*^trans^ value is equal to the permeability surface area product per unit volume of tissue [19]. NPC tumor tissues are characterized by extensive angiogenesis, an incomplete vascular basement membrane, and high permeability. Thus, NPC tumor tissues exhibit high permeability conditions, with leakage of CA to the EES being rapid and of great quantity. Accordingly, the *K*^trans^ value for NPC tissue is very high. In contrast, muscle tissue, fibrous tissue, normal brain tissue, and tumor tissues in the late stages of chemotherapy or radiation therapy are characterized by low permeability. The *K*^trans^ value for these tissue types is dependent on cross-vessel wall transport, and it is significantly lower than the *K*^trans^ value for tumor tissues [27, 28]. Swelling, degeneration, and necrosis of tumor capillary walls, as well as narrowing of vessel lumen and thrombosis due to radiotherapy, also contribute to decreased permeability and reduced perfusion, thereby resulting in a lower *K*^trans^ value. Many studies have reported on the prognostic value of *K*^trans^ and other DCE-MRI parameters in assessing the response to radiotherapy, which, however, demonstrated contrary findings. Longitudinal study and repeated DCE-MRI may reduce these discrepancies, and be recommended [29].

A decrease in the *K*^trans^ values for the NPC tumor tissues was observed significantly earlier (e.g., at the RT 50 Gy timepoint) in the combined treatment group than in the radiotherapy alone group (3 months post-RT). These results suggest that LMP1-targeted DNAzymes accelerate the decline of the *K*^trans^ value for NPC. It has previously been shown that EBV LMP1 promotes tumor angiogenesis by up-regulating VEGF expression via activation of Stat 3 transcriptional factor [3]. Although the relationship between DCE-MRI and VEGF remains unclear, a positive correlation between levels of soluble vascular endothelial growth factor recptor-1 (sVEGFR-1) and sVEGFR-2 in plasma and *K*^trans^ for patients with advanced ovarian cancer were observed in a previous study [30]. Taken together, these observations suggest that LMP1-targeted DNAzyme treatment affects the angiogenesis and microvascular permeability of NPC, and this can be proved with further pathology and molecular biology studies of NPC.

In cancer treatment trials, *k*_ep_ and *v*_e_ values have been recommended as secondary endpoints [31]. In the present study, tumor tissues of both the combined treatment group and the radiotherapy alone group had *k*_ep_ values that declined following therapy due to the slower transfer of CA from the EES to plasma as a result of decreased permeability. This similar trend in *k*_ep_ values for both groups suggests that the decrease is mainly related to the radiation treatments. In contrast, *v*_e_ values for all of the tumor tissues increased following radiotherapy, and this is consistent with an increase in EES secondary to the loss of tumor cells with radiation exposure. Moreover, continuous accumulation of CA in the EES is consistent with a slower transfer of CA from EES to plasma post-therapy [31, 32].

There were limitations associated with the present study. First, since surgery is not the preferred treatment for NPC, a comparison of pathology and DCE-MRI was not conducted. Secondly, the majority of the tumor lesions examined exhibited reduced tumor volume and disappeared quickly following treatment. Consequently, there was a lack of DCE-MRI data after the first year following treatment. Lastly, the parameters examined were based on the use of a tracer-kinetic model, which assumes that transcytolemmal water exchange contributes negligibly to changes in signal intensity [33]. Therefore, the amount of CA in tissues could be overestimated.

## Conclusions

The use of an EBV LMP1-targeted DNAzyme was found to accelerate the decline of *K*^trans^ values for NPC tissues, thereby suggesting that this DNAzyme affects the angiogenesis and microvascular permeability of NPC *in vivo*.

## Abbreviations

DCE-MRI: Dynamic contrast-enhanced magnetic resonance imaging
LMP1: Latent membrane protein 1
DNAzymes: Deoxyribozymes
NPC: Nasopharyngeal carcinoma
Pre-RT: 1 ~ 2 days before radiotherapy
RT 50Gy: When a radiation dose of 50 Gy was achieved
RT 70Gy: When a radiation dose of 70 Gy was achieved
3 months post-RT: 3 months after radiotherapy
AIF: Arterial input function
ROI: Region of interest
EES: Extravascular extracellular space
CA: Contrast agent
*K*^trans^: The volume constant for the transfer of contrast from plasma to the extravascular extracellular space
*k*_ep_: The rate constant for the transfer of CA from the EES to plasma
*v*_e_: CA distribution volume.
